# *Medinillatheresae* (Melastomataceae), a new species from ultramafic soils in the Philippines

**DOI:** 10.3897/phytokeys.113.30027

**Published:** 2018-12-11

**Authors:** Edwino S. Fernando, J. Peter Quakenbush, Edgardo P. Lillo, Perry S. Ong

**Affiliations:** 1 Department of Forest Biological Sciences, College of Forestry and Natural Resources, The University of the Philippines – Los Baños, College, 4031 Laguna, Philippines; 2 Institute of Biological Sciences, College of Arts and Sciences, The University of the Philippines – Los Baños, College, 4031 Laguna, Philippines; 3 Department of Forestry, Cebu Technological University, Argao, Cebu, Philippines; 4 Department of Biological Sciences, Western Michigan University, Kalamazoo, MI 49008-5200, USA; 5 Institute of Biology, College of Science, The University of the Philippines – Diliman, 1101 Quezon City, Philippines

**Keywords:** Dinagat Island, *Medinilla*, Melastomataceae, Mt Hamiguitan, ultramafic soils

## Abstract

A new species, *Medinillatheresae* Fernando, from ultramafic soils on Dinagat and Mindanao Islands, Philippines is described and illustrated. The species is characterized by its terrestrial erect habit, non-setose nodes, 3-plinerved, lanceolate and coriaceous leaves arranged in whorls, cauline or axillary and pendulous inflorescences, rounded flower buds, 4-merous flowers, and straight anthers. It is compared with other similar species in the *Medinillapendula* Merr. complex.

## Introduction

*Medinilla* Gaudich. (Melastomataceae) is a genus of terrestrial and epiphytic shrubs and climbers occurring from Tropical Africa, Madagascar, to India, Sri Lanka, Myanmar, southern China and Taiwan, throughout Southeast Asia, New Guinea, northern Australia, Micronesia, Solomons, Vanuatu, Fiji, and Samoa with about 375 species (Bodegom and Veldkamp 2001; Mabberley 2017). In the Philippines, Merrill (1913) early on attributed just 28 species, then later 125 species (Merrill 1923). In the revision of the Philippine species of this genus, Regalado (1995) recognized 80 species with 90% endemicity. These species were divided into 12 informal species groups based on a combination of indumentum, leaf, stem, and inflorescence characters, and within each group additional characters of the stem, leaf, inflorescence, and flower were used to delimit species.

Since Regalado’s (1995) revision only one species, *Medinilladallciana* Fernando & Balete (Fernando and Balete 2013) belonging to Group 5, has been added to the Philippine list.

In this paper, we describe a new species, *Medinillatheresae*, a terrestrial, erect, cauliflorous shrub from ultramafic soils on Dinagat and Mindanao Islands, Philippines.

## Materials and methods

This new species of *Medinilla* was discovered while undertaking a field survey of the ultramafic flora of Dinagat Island. Photographic records were taken and herbarium specimens collected. The morphological description of the species is based on vegetative and reproductive characters. Field characters were recorded on site; vegetative characters were observed and measured from press-dried specimens and seedlings and reproductive characters from fresh specimens and from material preserved in 70% ethanol. All morphological measurements were made using digital calipers and a calibrated eye piece under a dissecting microscope. Herbarium specimens were also examined and compared at CAHUP, CMUH, LBC, MO, PNH, and PUH, including additional material, e.g. images of type specimens of Philippine *Medinilla* available online at A, CAS, GH, K, L, NY, UC, and US. All photographs, except where indicated, were taken in the field in the natural habitat of the species.

## Taxonomy

### Medinilla theresae

Taxon classificationPlantaeMyrtalesMelastomataceae

Fernando sp. nov.

urn:lsid:ipni.org:names:60477661-2

**Figure 1.**
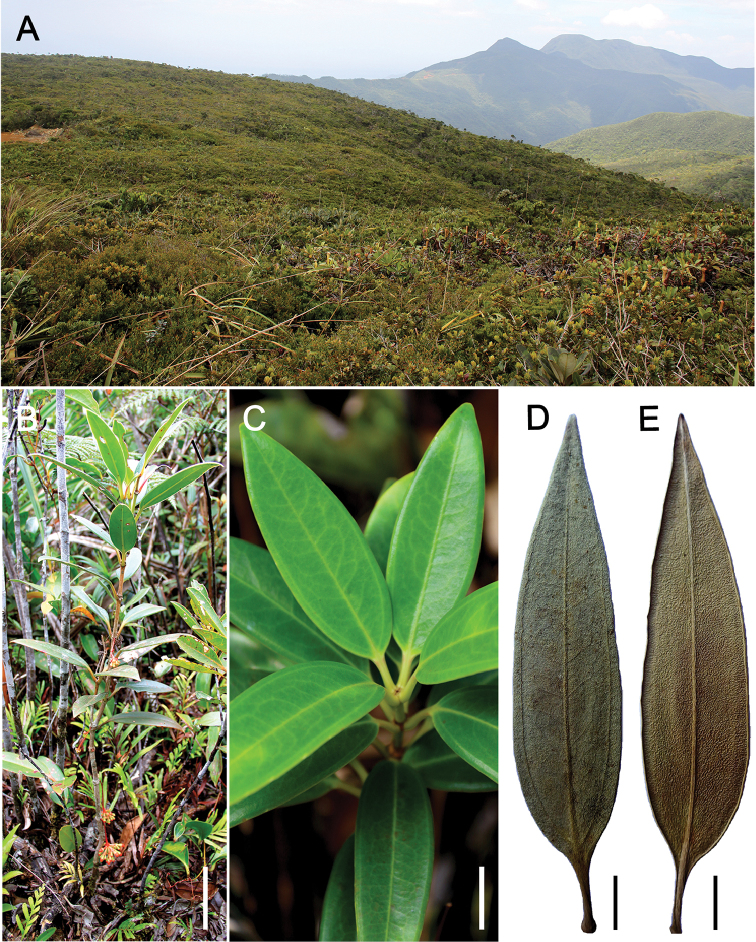
*Medinillatheresae* Fernando **A** Habitat at type locality, dwarf forest on ultramafic soils, *c.* 840 m elevation, Mt Redondo with Mt Kambinliw in the background **B** Terrestrial, erect growth habit **C** Leafy branch showing leaves arranged in a whorl and secondary veins faintly visible on adaxial surface **D** Dried leaf showing adaxial surface with distinct pair of secondary veins **E** Abaxial surface of same leaf in D without the distinct pair of secondary veins. **B, D, E** from *Fernando 3831* (LBC), **C** from *Fernando 4166* (LBC). Scale bars: 10 cm (**B**); 2 cm (**C–E**). All photos by Edwino S. Fernando.

**Figure 2.**
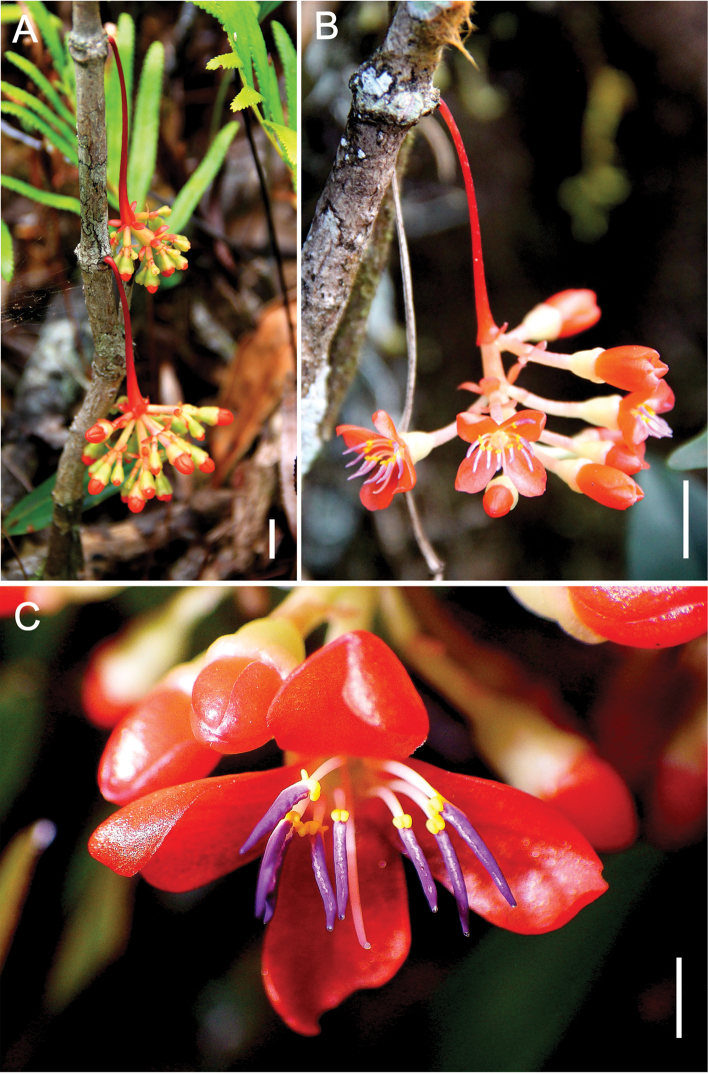
*Medinillatheresae* Fernando **A** Pendulous inflorescences arising from nodes near base of main stem and showing flower buds with rounded tips **B** Inflorescence with buds and open flowers **C** Close up of open flower. **A** from *Fernando 4166* (LBC) **B, C** from *Fernando 3831* (LBC). Scale bars: 1 cm (**A**); 8 mm (**B**); 2 mm (**C**). All photos by Edwino S. Fernando.

**Figure 3.**
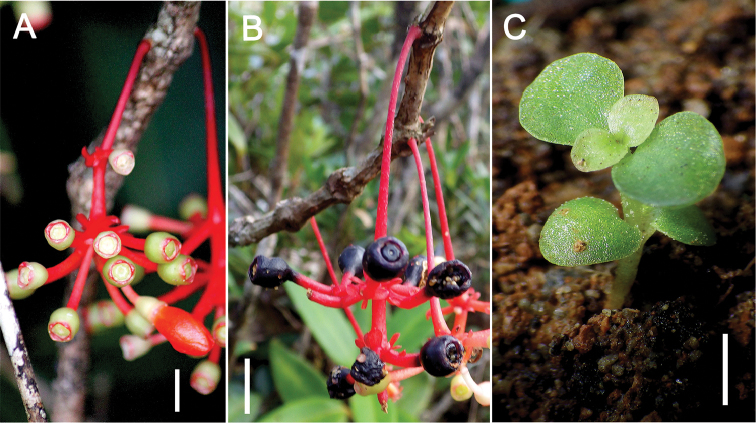
*Medinillatheresae* Fernando **A** Young infructescence showing light green fruits with bright red calyx rim **B** Mature purplish-black fruits **C** Seedling showing foliaceus cotyledons and first two pairs of eophylls, *c.* 20 weeks old, grown in nursery from seed of *Fernando 3831*. **A** from *Fernando 4217* (LBC) **B** from *Fernando 3831* (LBC). Scale bars: 1 cm (**A, B**); 2 mm (**C**). All photos by Edwino S. Fernando.

**Figure 4.**
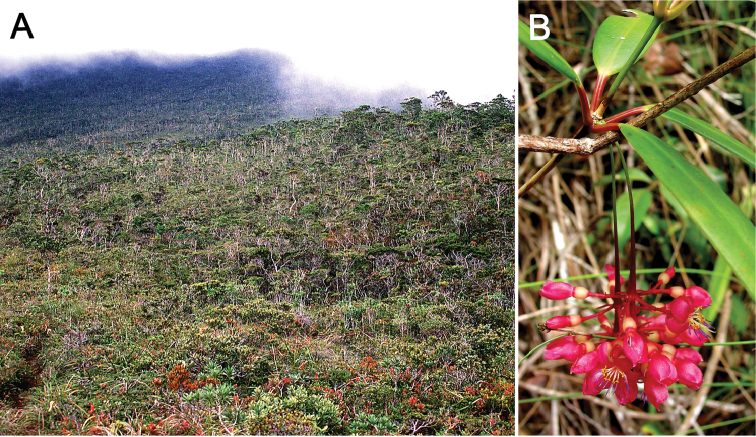
*Medinillatheresae* Fernando **A** Habitat on Mt Hamiguitan, forest on ultramafic soils, *c.* 1200 m elevation **B** Plant on Mt Hamiguitan with branch showing node with four leaves in a whorl and a pair of pendulous inflorescences. **A** photo by Edwino S. Fernando **B** photo by Leonard L. Co.

Figures 1 , 2 , 3 , 4

#### Diagnosis.

This species is most similar to the *Medinillapendula* species complex in its whorled leaves, 4-merous flowers, and pendulous inflorescences. It differs, however, in its secondary veins of leaves being distinct only on the adaxial surface, cauline or axillary inflorescences, and straight anthers.

#### Type.

PHILIPPINES. Dinagat Island: Municipality of Loreto, Mt Redondo, 10°35'34.2"N, 125°63'49.0"E, 840 m elevation, dwarf forest on ultramafic soil, flower buds and open flowers, 29 September 2015, *E.S. Fernando 3831* (holotype PNH; isotypes LBC, PUH).

#### Description.

Terrestrial, erect *shrub* up to 1.5 m tall. *Stem* 1−2 cm diameter near the base of the plant, terete, internodes to 12 cm long, shorter on the distal branches; nodes rather thickened, knobby, not setose, although small barbules may sometimes appear on younger nodes; bark generally smooth, becoming striate on older stems; young stems terete, about 3 mm in diameter, green. *Leaves* simple, petiolate, in whorls of 3 or 4 per node; petiole ascending, about 3−5 mm thick, 0.5−2 cm long, pale light green, sometimes with dark purplish-red or maroon tinge on the adaxial side; lamina lanceolate, 5−12 × 1.5−4 cm, glossy dark green adaxially, paler abaxially, succulent when fresh, coriaceous when dry; base obtuse, apex acuminate; 3-plinerved, the pair of secondary veins diverging about 2−5 mm from the leaf base, in fresh specimens only visible on the adaxial surface, very faintly so and only near the leaf base on the abaxial surface, in dry specimens visible only on the adaxial surface; transverse veins faintly visible on adaxial surface in fresh and dry specimens, indistinct or absent abaxially; margins smooth, revolute at the edges. *Inflorescences* cauline, not terminal, arising from leafless nodes, sometimes near the base of the stem, or from leafy nodes, pendulous, usually solitary, or sometimes two or three per node; peduncle about 2.5−6 cm long, enlarged towards the distal end, bright red at maturity, bracteate, each bract 4 × 3 mm; flowers up to 15 or more per inflorescence, usually clustered in a whorl of short, 3-flowered cymes only at the enlarged, distal end of the peduncle, sometimes in 2−3 whorls; 10 or so arranged in umbellately cymose clustered branches up to about 1 cm long, also subtended by bracts; secondary bracts spatulate, 3 × 2 mm; total inflorescence length about 7 cm. *Flower buds* ± rounded at the tips, the petals imbricate. *Flowers* 4-merous, petal 8 × 4 mm, oblique-oblong, often reflexed, orange-red, red, or pink; stamens 8, usually positioned above the style, anthers linear-lanceolate, 2.5−4 mm long, rather straight, purple, with a yellow dorsal spur of 0.6 mm long on the connective and a pair of partly joined stout, ventral appendages at the base of the anther sac; filament 4 mm long, pale white; style terete, 10 mm long, pale white; hypanthium campanulate, the rim generally truncate or sometimes very shallowly lobed, pale or light green, 3−4 × 3−4 mm; pedicel pale pink or red, about 7 mm long. *Fruit* a subglobose berry, 4−6 × 5−7 mm, light green when young and with bright red calyx rim, entirely purplish-black when ripe; the peduncle and pedicels red. *Seeds* numerous, embedded in pulpy tissue, ovoid, 1−1.5 × 0.5−1 mm, chestnut brown. *Seedling* with epigeal germination, phanerocotylar, cotyledons foliaceus, 2−3 × 2 mm, broadly ovate, apex rounded or obtuse, sometimes shallowly emarginate; eophylls simple, opposite, broadly elliptic-ovate to orbicular, 3−7 × 3−5 mm.

#### Additional specimens examined.

PHILIPPINES. Dinagat Island, Municipality of Loreto, Mt Redondo, 10°35'06.3"N, 125°63'03.6"E, 700 m elevation, flower buds, 1 September 2016, *Fernando 4166* (LBC, PNH, PUH); 700 m elevation, flowers, 30 September 1991, *Gaerlan, Sagcal, & Fernando PPI 4651* (MO [MO5547927], PNH); 10°35'19.3"N, 125°63'24.2"E, 800 m elevation, juvenile fruits, 1 September 2016, *Fernando & Matute 4217* (LBC). Mindanao Island, Pujada Peninsula, Davao Oriental Province, Municipality of San Isidro, Mt Hamiguitan, 900 m elevation, flowers, 25 February 2005, *Amoroso & Aspiras CMUH 04922* (CMUH); 6°44'16.728"N, 126°10'1.02"E, 1326 m elevation, fruits, 23 June 2015, *Fritsch et al. 2025* (CAS [CAS493220], CMUH). Cultivated: Luzon Island, Laguna Province, Municipality of Los Baños, seedlings grown from seeds of *Fernando 3831* germinated in nursery, 4 May 2016, *Fernando 3831A* (LBC).

Some photos of Leonard L. Co from Mt Hamiguitan taken in January 2005 and appearing in PhytoImages (www.phytoimages.siu.edu) with five of these identified as *Medinillasurigaoensis* (DOL nos. 27282−27284, 27439, and 27534) and several others as *Medinilla* sp. (DOL nos. 27278−27281 and 27533) (Pelser et al. 2011) belong to this new species. Unfortunately, we have been unable to find at PUH, where Mr Co was based, the specimens or his field notes associated with the photos. The vouchers are likely among approximately 6,000 unprocessed specimens still wrapped in about 200 plastic bags.

#### Habitat and ecology.

On Mt Redondo, Dinagat Island, this species occurs in dense, dwarf forest 1−2 m tall on ultramafic soils on gentle slopes at *c.* 700−840 m elevation (Figure 1A). This dwarf forest is about 527 hectares (5.27 km^2^) with more than 90% of the trees having stem diameter of less than 10 cm (Fernando et al. 2017). The rhizospheric soil (*c.* 30 cm) here reveal, on average, up to 396,024 ppm iron (Fe); 1,344 ppm nickel (Ni); 425 ppm copper; 10,875 ppm chromium; and 4,453 ppm manganese (elemental analyses obtained by x-ray fluorescence, unpublished data). On Mt Hamiguitan, it occurs at the edges of the upper montane forest at 900 m elevation, up to the so-called ‘mossy-pygmy forest’ at 1160−1200 m and 1460−1600 m elevation (Figure 4A), also on ultramafic soils, where the trees are 0.5−2.5 m tall and with an average stem diameter of 8 cm and dominated by species of *Leptospermum* (Myrtaceae), *Weinmannia* (Cunoniaceae), *Elaeocarpus* (Elaeocarpaceae), and *Dacrydium* (Podocarpaceae) (Amoroso and Aspiras 2011), and also including the heavy metal indicator *Scaevolamicrantha* C.Presl (Goodeniaceae) (Fernando et al. 2008; Amoroso and Aspiras 2011).

The small trees and shrubs associated with *Medinillatheresae* at the type locality on Dinagat Island include, among many others, *Leptospermumamboinense* Blume (Myrtaceae), *Psychotriasurigaoensis* Sohmer & A.P.Davis (Rubiaceae), *Scaevolamicrantha*, *Rhodomyrtussurigaoensis* Elmer (Myrtaceae), *Calophyllumcucculatum* Merr. (Calophyllaceae), *Ternstroemiagitingensis* Elmer (Pentaphylacaceae), *Fagraeagitingensis* Elmer (Gentianaceae), *Gardeniabarnesii* Merr. (Rubiaceae), *Timoniusvaletonii* Elmer (Rubiaceae), *Dacrydiumbeccari* Parl. (Podocarpaceae), *Falcatifoliumgruezoi* de Laub. (Podocarpaceae), and various species of *Syzygium* (Myrtaceae). There are also vines such as *Dischidiamajor* (Vahl) Merr. (Apocynaceae), *Flagellariaindica* L. (Flagellariaceae), *Nepenthesmindanaoensis* Sh.Kurata (Nepenthaceae), and species of *Freycinetia* (Pandanaceae).

#### Distribution.

Thus far, this new species is known only from Mt Redondo on Dinagat Island and Mt Hamiguitan in the Pujada Peninsula on Mindanao Island, Philippines. Dinagat Island, Surigao del Norte Province, and the Pujada Peninsula form part of the same belt of the Eastern Philippine Cretaceous ophiolite and ophiolite complexes (Balce et al. 1976; Yumul et al. 2003, 2008; Tamayo et al. 2004) that are now large areas of ultramafic landscapes with metallic ore deposits (e.g., iron, nickel, chromium) and hosting a unique type of forest formation (Fernando et al. 2008).

#### Etymology.

This beautiful new species is named in honor of Dr Theresa Mundita S. Lim, former Director of the Biodiversity Management Bureau, Department of Environment and Natural Resources of the Philippines, and now Executive Director of the ASEAN Centre for Biodiversity, whose dedication and commitment to protecting Philippine biodiversity is admirable. Director Lim has also been active in the international biodiversity conservation sector.

#### Notes.

In the glabrous nature of the plant and whorled leaves, this new species belongs in Group 1 of Regalado (1995) being most similar to *Medinillapendula* Merr. (Merrill 1905) under which Regalado (1995) reduced to synonymy four species viz., *Medinillaelmeri* Merr. from Mt Sto. Tomas, Benguet Province, Luzon Island (Elmer 1911); *Medinillasubsessilis* Merr. from Melamey, Bontoc, Luzon Island (Merrill 1912); *Medinillagitingensis* Elmer from Mt Giting-giting, Sibuyan Island (Elmer 1911); and *Medinillamerrillii* Elmer from Mt Apo, Mindanao Island (Elmer 1911). Of these, our new species is most similar to *Medinillamerrillii* in its non-setose nodes and 3-plinerved leaves, but differs in its terrestrial, erect habit, leaf secondary veins distinct on adaxial side only, cauline inflorescences arising from leafless nodes, shorter inflorescence, and straight anthers. It is also similar to *Medinillagitingensis* in its terrestrial, erect habit, non-setose nodes, and 3-plinerved leaves, but differs in its leaf secondary veins distinct on adaxial side only, shorter cauline and sometimes axillary inflorescences, rounded flower buds, and straight anthers (see Table 1). *Medinillatheresae* can be readily distinguished by the combination of whorled leaves and non-terminal pendulous inflorescences.

**Table 1.** Diagnostic characters separating *Medinillatheresae* from species in the *Medinillapendula* complex.

	* Medinilla theresae *	* Medinilla merrillii *	* Medinilla gitingensis *	*Medinillapendula (s.str.)*
Stem diameter (cm)	1−2	3	7.5	–
Nodes	not setose	not setose	not setose	setose
Number of leaves per node	3−4	several, 3−5	3	4 or 5
Petiole (mm)	5−20	17−20	15−25	10−15(−20)
Lamina shape	lanceolate	narrowly elliptic	narrowly elliptic	narrowly elliptic
Leaf venation (based on dried specimens)	3-plinerved; secondary veins distinct on adaxial side only	3-plinerved; secondary veins distinct on both sides	3-plinerved; secondary veins distinct on both sides	generally 5-, rarely7-plinerved; secondary veins distinct on both sides
Inflorescence position	cauline, not terminal, arising from leafless or leafy nodes	axillary	terminal	terminal, sometimes axillary
Inflorescence length (cm)	7	20	10−20	12−25
Flower in bud	± rounded	± rounded	pointed	± rounded
Anthers	straight	curved	curved	curved

The cauline and many-flowered inflorescences also puts this new species in Regalado’s (1995) Group 5 where it is most similar to *Medinillaaurantiflora*Elmer (1911) from Negros, Panay, and Sibuyan Islands in its erect habit and 4-merous flowers, but our new species is distinguishable by its ternate to quaternate smaller leaves, shorter hypanthia, and straight anthers. The combination of terrestrial erect habit and cauline, many-flowered inflorescences is also known in *Medinillalagunae* S.Vidal and in *Medinillaphilippensis* (Cham. & Schtdl.) Merr., the latter was synonymized by Regalado (1995) under *Medinillavenosa* (Blume) Blume but recommended by Quakenbush (2016) to be reinstated as a distinct species. In all these species, the leaves are 7−11-plinerved, while in *Medinillatheresae* they are 3-plinerved. Elsewhere in Southeast Asia, the terrestrial erect habit and cauline, many-flowered inflorescences is also known in *Medinillatapete-magicum* Cámara-Leret & Veldk. (Cámara-Leret and Veldkamp 2011) from Sulawesi. However, in this species the flowers are 5-merous and borne on very compact inflorescences arising from the stem at or near ground level forming a dense mat around the base of the plant.

Two other species of *Medinilla* also grow in the Mt Redondo area of Dinagat Island (Fernando et al. 2017). At about the same elevation as the new species described here is one referable to *Medinillamyrtiformis* (Naudin) Triana, an epiphytic shrub with prominently divaricate branches and opposite leaves, widespread in most upper montane rain forests in the Philippines and is also known from Sulawesi and Moluccas (Regalado 1995). On the lower slopes of the mountain at 300−650 m elevation is the scandent *Medinillaquadrifolia* (Blume) Blume, regarded as a widespread and highly variable, polymorphic species (Regalado 1995).

In the Surigao del Norte area, at low elevations (*c.* 150 m), also on ultramafic soils, ternate to quaternate and 3-plinerved leaves are also known in *Medinillasurigaoensis*Regalado, a species belonging to Group 6 (Regalado 1995). However, this species is an epiphytic scandent shrub and has generally smaller leaves that are ovate to elliptic-obovate and with secondary veins distinct on both surfaces of the lamina, sulcate branchlets, and the fruits have prominent outgrowths or protuberances on the surface. It is uncertain if this species is an ultramafic endemic as Regalado (1995) also recorded it from Luzon and Polillo Islands.

*Medinillapalawanensis* Regalado was earlier described as an edaphic endemic on ultramafic rock slopes on Mt Beaufort near Puerto Princesa in Palawan (Regalado 1995). However, since then this epiphytic shrub has been found on nearby Mt Cleopatra, not an ultramafic site (J.P. Quakenbush, personal observation). *Medinillacapitata* Merr. and *Medinillaferruginea* Merr. were both originally described from ultramafic soils at low elevations on Dinagat Island (Merrill 1920). *M.capitata* has now also been recorded from likely an ultramafic site in Samar (Pelser et al. 2011) and there are additional herbarium records in PNH. Similarly, for *M.ferruginea* there are specimens at PUH (from Surigao, Mindanao) and MICH (from Camarines, Luzon), as well as, records from Pelser et al. (2011) in southern Samar that may possibly correlate with ultramafic soils. Further field work in these sites is needed to confirm the restriction of these two species to such edaphic conditions. *M.gitingensis*, although presently treated as *M.pendula*, was only known from an ultramafic location. No Philippine species of *Medinilla* are otherwise known by us to be endemic to ultramafic soils.

#### Conservation status.

*Medinillatheresae* is an edaphic-endemic, thus far restricted to forests on ultramafic soils at elevations of *c.* 700−1326 m. The species is known only from two locations, Mt Redondo and Mt Hamiguitan, over 400 km apart. Using the online GeoCAT conservation assessment tool (http://geocat.kew.org/) with the default 2 × 2 km grid calculated an EOO (extent of occurrence) of more than 100 km^2^ but less than 5,000 km^2^ and AOO (area of occupancy) of 16 km^2^ which are thresholds for the Endangered category (IUCN 2012). Following IUCN (2012) and the *Guidelines for Using the IUCN Red List Categories and Criteria* (IUCN Standards and Petitions Subcommittee 2017), we regard this species as Endangered [EN B1+2ab(ii,iii,v)]. In both the two currently known locations of this species a continuing decline is inferred in area of occupancy, the area, extent and/or quality of habitat, and number of mature individuals. On Mt Redondo, the dwarf forest is within a mineral reservation that was previously subject to open pit mining for the heavy metal chromium. If mining is allowed to continue here, the species will be at high risk. Mt Hamiguitan, on the other hand, is a declared protected area, formally known as the Mt Hamiguitan Range Wildlife Sanctuary (MHRWS) and is a UNESCO World Heritage Site which may provide the species with some protection. MHRWS is popular site for nature trekking enthusiasts, but some hiking trails lead through the pygmy forest where this species occurs.

## Supplementary Material

XML Treatment for Medinilla theresae
